# Graphdiyne nanosheets as a platform for accurate copper(ii) ion detection *via* click chemistry and fluorescence resonance energy transfer†

**DOI:** 10.1039/d0ra08595b

**Published:** 2021-01-28

**Authors:** Chenchen Ge, Jiaofu Li, Dou Wang, Kongpeng Lv, Quan Liu, Yan Shen, Xiaoqing Zhuang, Wankun Luo, Zongze Wu, Yuhua Zhang, Lulin Shi, Liping Liu, Shiyun Bao, Han Zhang

**Affiliations:** Department of Hepatobiliary and Pancreatic Surgery, The Second Clinical Medical College, Jinan University (Shenzhen People's Hospital) Shenzhen 518020 China bao.shiyun@szhospital.com liu.liping@szhospital.com; Institute of Microscale Optoelectronics, Collaborative Innovation Centre for Optoelectronic Science & Technology, Key Laboratory of Optoelectronic Devices and Systems of Ministry of Education and Guangdong Province, Guangdong Laboratory of Artificial Intelligence and Digital Economy (SZ), Shenzhen University Shenzhen 518060 PR China hzhang@szu.edu.cn; College of Health Science and Environmental Engineering, Shenzhen Technology University Shenzhen 518118 China; Department of Biomedical Engineering, Southern University of Science and Technology Shenzhen 518055 China

## Abstract

A novel sensing platform for sensitive detection of copper(ii) ions (Cu^2+^) in living cells and body fluids was developed by taking advantage of the excellent fluorescence quenching ability of graphdiyne (GDY) and the high specificity of click chemistry for the first time.

## Abstract 2

Cu^2+^ detection was performed by taking advantage of the fluorescence quenching ability of graphdiyne and the high specificity of click chemistry.

## Graphical abstract

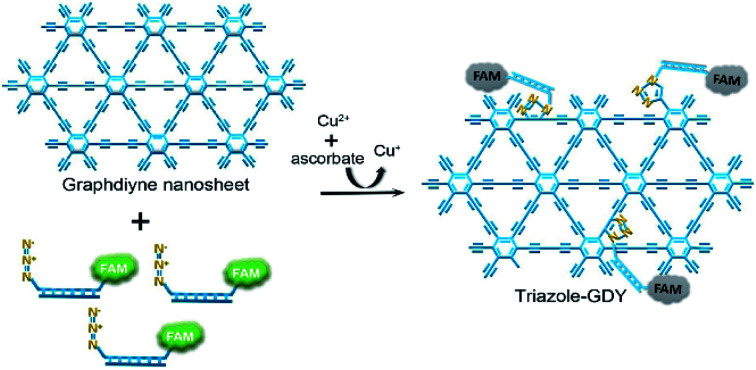

Cu^2+^ is an essential element which acts as a ligand of various redox enzymes and metalloproteins used in metabolic processes for human life.^1^ These enzymes and proteins, such as tyrosinase, cytochrome C oxidase, dopamine β-hydroxylase, and ceruloplasmin, play important roles in electron transfer, dioxygen binding, activation, and reduction.^2^ As a consequence, the concentration of Cu^2+^ in living systems should be strictly regulated and its early diagnosis is required for controlling the occurrence and development of Cu^2+^ related diseases. For example, a relatively high level of Cu^2+^ in urine is one of the clinical and diagnostic indicates of Wilson's disease, where screening of the disease *via* Cu^2+^ detection in patients' urine at early stage can greatly improve their cure rate and life quality.^3^ Therefore, developing a convenient and sensitive method to monitor Cu^2+^ in living cells or biological fluids is extremely vital for both scientific research and clinical diagnostics.

Up till now, considerable strategies have been developed for Cu^2+^ detection based on colorimetric, electrochemical, fluorescence, *etc.*^4–6^ Among these methods, only fluorescence assay can satisfy the demand of intercellular imaging, which can give more critical information for fundamental research. Currently, a majority of fluorescent sensors for Cu^2+^ imaging are based on synthetic organic molecules.^7^ However, the structure of organic molecules should be strictly design to obtain tight binding affinity to Cu^2+^. Meanwhile, these molecules also require rigorous synthetic procedures and purifications, which limits their wide application. In the past decade, low dimensional nanomaterials have been exploited as nanoquenchers to fluorescent dyes, which were based on the mechanism of förster resonance energy transfer (FRET).^8^ Among them, two-dimensional (2D) nanomaterials, such as carbon nanomaterials,^9,10^ MXenes,^11^ and black phosphorus,^12^ have been demonstrated to be excellent platforms for quenching fluorescent dyes. This is because 2D nanomaterials possess planar structure and ultra-large surface area, which enable them easily adsorb biomolecules while maintaining maximum quenching efficiency to fluorescent dyes than other low-dimensional nanomaterials, such as gold nanoparticles, quantum dots, nanorods, and nanowires.^8^

Graphdiyne nanosheets is a 2D carbon nanomaterial with sp- and sp^2^-hybridized carbon atoms. Graphdiyne (GDY), a member in graphyne family that consists of diacetylene groups and benzene ring, was first proposed by Haley,^13,14^ and it is the only graphyne member which can be synthesized in large scale. The synthesis of GDY is through an *in situ* cross-coupling reaction using hexaethynylbenzene on the surface of copper which was first reported by Li's group in 2010.^15^ However, both the kinetic and thermodynamic stabilities of GDY are inferior to those of graphene. Kinetically, the triple bonds of GDY are more chemically reactive than the aromatic bonds of graphene. Thermodynamically, GDY is also less stable than graphene because the sp carbons of GDY are more chemically unsaturated. The unique structure of GDY have attracted extensive attention in both fundamental researches and practical applications, and GDY has been demonstrated to be successfully applied in catalysis,^16^ energy storage,^17^ electronic devices,^18^ water purification,^19^ detector,^20^ and cancer theranostic.^21^ Similar to graphene, GDY NSs not only adsorbed single-stranded DNA (ssDNA) probe instead of double-stranded DNA (dsDNA) *via* van der Waals force and π–π stacking interaction between nucleobases and GDY, but also possessed fluorescence quenching properties due to FRET between dye molecule and hexagonal cells of GDY NSs.^22^ GDY is a better fluorescence quencher than graphene through theoretical calculations which was proved by Nargish Parvin *et al.* in 2017.^23^ The density functional theory calculation shows that the interaction between GDY and the dye molecular FAM is stronger than that of graphene. The calculation results of the partial density of states confirm that the presence of GDY's acetylene consisting of two π bonds in the three bonds facilitates the ssDNA adsorption, which leads to a further enhancement of the effective fluorescence quenching.

In recent years, copper(i) ion (Cu^+^)-catalyzed click chemistry has drawn great attention due to its high efficiency and selectivity. Click chemistry, which was first proposed by Sharpless *et al.* in 2001, refers to the reaction between an azide group (–N_3_–) and an alkyne group (–C≡C–) in the presence of Cu^+^, resulting the formation of a five-membered triazole ring.^24^ The source for Cu^+^ in the click chemistry can be generated from the reduction of Cu^2+^ in the presence of sodium ascorbate (SA). The high efficiency of click reactions can occur under physiological conditions and virtually free of side reactions even in the presence of cells, cell lysates, or biological fluids.^25^

By taking advantages of the excellent fluorescence quenching ability of GDY and the high specificity of click chemistry, we proposed an approach for accurate and sensitive Cu^2+^ detection in the present study. Fig. 1 illustrates the schematic representation of Cu^2+^ sensing platform. Mechanically, the N_3_ groups of N_3_-dsDNA-6-carboxyfluorescein (N_3_-dsDNA-FAM) were specifically conjugated with the butadiyne groups of GDY NSs through click chemistry in the presence of Cu^2+^ and SA, resulting stable five-membered triazole rings formation, denoted as triazole-GDY. The close proximity of FAM and GDY NSs facilitated the FRET from FAM to GDY NSs, which resulted in fluorescence quenching of FAM. The Cu^2+^ concentration was monitored by the change of fluorescence intensity (FL intensity). The application of this biosensor was further confirmed in living cells and human urine samples.

**Fig. 1:**
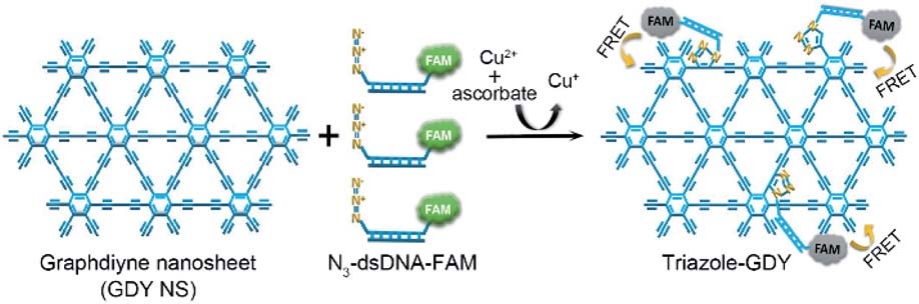
Schematic illustration of GDY NSs sensing platform for sensitive fluorescence detection of Cu^2+^ based on Cu^+^-catalyzed click chemistry.

GDY NSs were firstly synthesized according to the previously reported method.^13^ The morphology of GDY NSs was characterized by transmission electron microscopy (TEM) and high-resolution (HR)-TEM. TEM images showed that the synthesized GDY had satisfactory morphology and flaky degree (Fig. S1†) and curved streaks in the HR-TEM image with the lattice parameter of 0.37 nm is assigned to the interlayer space of GDY NSs (Fig. 2A). Raman spectrum (Fig. 2B) of GDY NSs showed four prominent Raman bands, where the bands centered at 1399.6 cm^−1^ and 1585.8 cm^−1^ are corresponded to the typical D and G band of carbonaceous materials, and the bands centered at 1932.6 cm^−1^ and 2170.7 cm^−1^ are corresponded to the typical vibrations of conjugated diynes. All of the above results demonstrated that GDY NSs were prepared successfully.

**Fig. 2:**
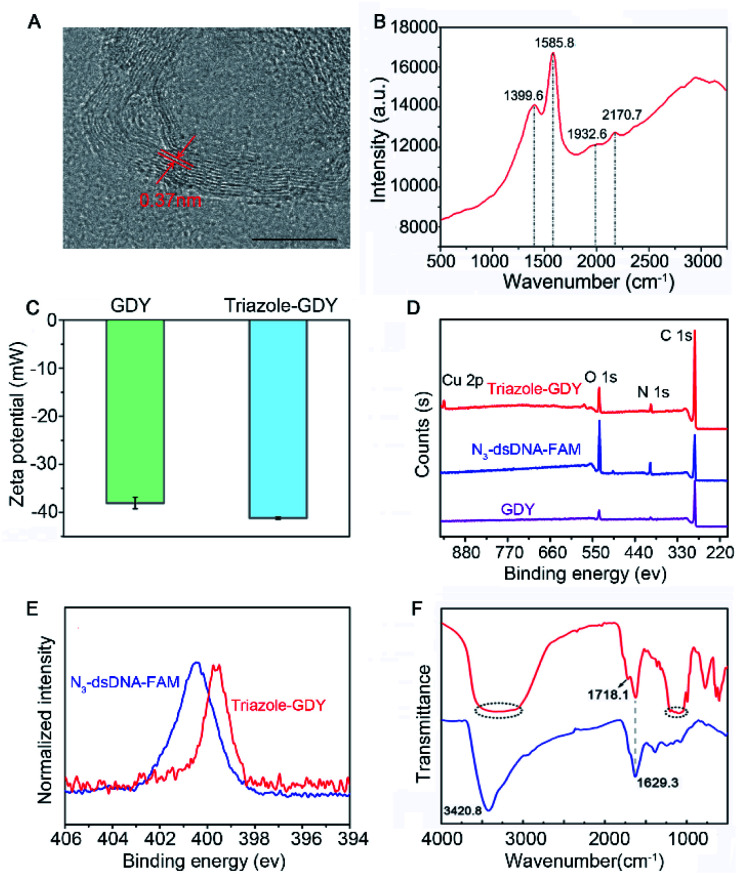
Characterization of GDY NSs and triazole-GDY. (A) HR-TEM image and (B) Raman spectrum of GDY NSs. Scale bar: 10 nm. (C) Zeta potentials of GDY NSs and triazole-GDY. (D) XPS spectra of GDY NSs, N_3_-dsDNA-FAM and triazole-GDY. (E) High-resolution N 1s spectra of N_3_-dsDNA-FAM and triazole-GDY. (F) FTIR spectra of GDY NSs and triazole-GDY.

The formation of triazole-GDY was further validated by zeta potential, X-ray photoelectron spectroscopy (XPS) and Fourier transform infrared spectrum (FTIR), respectively. The zeta potentials (Fig. 2C) changed from −38.1 mV to −41.2 mV after click reaction, which attribute to the formation of triazole ring and the ligation of negatively charged dsDNA-FAM to the surface of GDY NSs. As shown in XPS survey spectra (Fig. 2D), the peaks at 398.5 eV were corresponding to the N1s of triazole-GDY and N_3_-dsDNA-FAM, respectively. Moreover, the high-resolution N 1s spectra were also recorded and distinctly different N 1s spectra were observed (Fig. 2E). Because of the strong π–π interaction between GDY and dsDNA-FAM, the N1s peaks of triazole-GDY shifted to lower binding energy, which confirmed the formation of triazole ring *via* click reaction. In addition, the FTIR spectra (Fig. 2F) also display three new peaks after click reaction. The peak at 1718.1 cm^−1^ and the broad peak from 1048.6 cm^−1^ to 1213.1 cm^−1^ correspond to the C=O stretching and C–O–C stretching of FAM, the broad peak from 3062.8 cm^−1^ to 3508.1 cm^−1^ correspond to the N–H stretching of DNA. These results confirmed that N_3_-dsDNA-FAM were specifically conjugated with the butadiyne groups of GDY NSs through click chemistry.

To further verify triazole-GDY formation is indispensable to FRET from FAM to GDY NSs, we measured the FL intensity change after incubation N_3_-dsDNA-FAM with Cu^2+^, SA, GDY, GDY/SA, SA/Cu^2+^, and GDY/SA/Cu^2+^, respectively. The obvious FL intensity decreasing can be observed in the GDY/SA/Cu^2+^ group (Fig. 3A), while other groups did not undergo click reaction showed no significant fluctuation in FL intensity, indicating that this assay for Cu^2+^ detection is feasible.

**Fig. 3:**
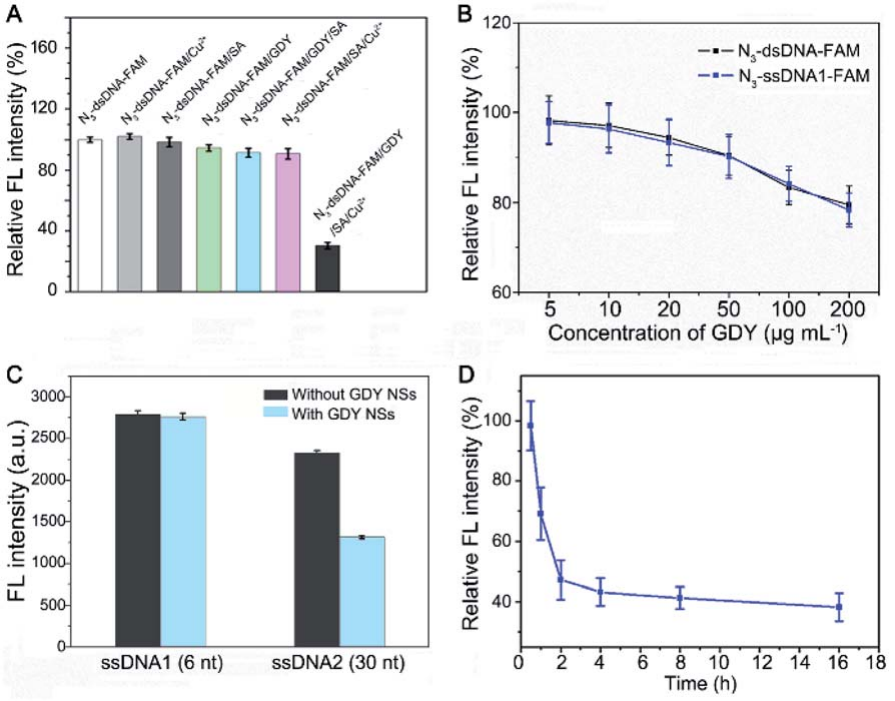
The feasibility of this assay and optimization of the experimental parameters. (A) The FL intensity change after incubation N_3_-dsDNA-FAM with Cu^2+^, SA, GDY, GDY/SA, SA/Cu^2+^, and GDY/SA/Cu^2+^, respectively. (B) Effect of the GDY NSs concentration on the initial FL intensity for Cu^2+^ detection. The concentration of N_3_-dsDNA-FAM and N_3_-ssDNA1-FAM was 10 nM. (C) The FL intensity of short and long strand N_3_-ssDNA-FAM after mixing with GDY NSs in PBS. (D) Effect of the click time on the FL intensity. The concentration of Cu^2+^ was 1 μM. The error bars represent the standard deviation of three independent measurements. Relative FL intensity is *F*/*F*_0_, where *F* is the FL intensity of the experimental group and *F*_0_ is the FL intensity of PBS.

Meanwhile, we found that the FL intensity of FAM is quenched under acidic condition and increased with pH value (Fig. S2†). In consideration that subsequent experiments need to be performed in living cells and body fluids, the neutral reaction buffer was selected (pH 7.4) in this assay.

Next, the ratio of GDY NSs and N_3_-dsDNA-FAM, which is closely related to the initial FL intensity and the sensitivity of this biosensor was investigated. If the amount of GDY NSs is insufficient, it cannot provide enough butadiyne groups for click reaction. On the contrary, the high concentration of GDY NSs in the solution shortens the distance between FAM groups and the surface of GDY NSs, resulting in the fluorescence quenching of FAM without click reaction. Accordingly, a set of GDY NSs concentrations (5 μg mL^−1^, 10 μg mL^−1^, 20 μg mL^−1^, 50 μg mL^−1^, 100 μg mL^−1^, and 200 μg mL^−1^) were investigated in the presence of 10 nM N_3_-dsDNA-FAM. The FL intensity showed no obvious fluctuation when the concentration of GDY NSs in the range of 5–10 μg mL^−1^, and the FL intensity decreased significantly with the increasing of GDY NSs concentration (Fig. 3B). Therefore, 10 μg mL^−1^ GDY NSs and 10 nM N_3_-dsDNA-FAM were used as the optimal concentrations for Cu^2+^ detection.

It has been reported that GDY NSs can adsorb and quench the fluorescence of ssDNA probe modified dye rather than dsDNA through FRET. Interestingly, it was found that the fluorescence of short strand N_3_-ssDNA1-FAM is almost the same as N_3_-dsDNA-FAM after mixing with GDY NSs in PBS (Fig. 3B), while the fluorescence of long strand N_3_-ssDNA2-FAM can be quenched significantly (Fig. 3C). This phenomenon may due to that the van der Waals force and π–π stacking interactions between GDY NSs and the six nucleobases (TTTTTT) of N_3_-ssDNA1-FAM is too weak to quench the fluorescence. The DNA sequences used in this experiment were listed in Table S1.† In order to measure the reaction rate of this Cu^2+^ detection system, the FL intensity of the solution at different time intervals was measured. Fig. 3D exhibits the effect of click time on the FL intensity in the presence of 1 μM Cu^2+^. The FL intensity decreased gradually with the extension of the reaction time. In consideration of the practical application of this biosensor, a suitable reaction time should be chosen. Consequently, we compromise the reaction time and 2 h was chose as the click time in this assay.

To evaluate the sensitivity and dynamic range of this fluorescence sensing platform for Cu^2+^ detection, a range of concentrations of Cu^2+^ (0, 0.01, 0.05, 0.1, 0.5, 1, 10, and 100 μM) were examined under optimal experimental conditions. As shown in Fig. 4A, the FL intensities at 518 nm of the reaction solution decreased gradually along with the increase of Cu^2+^ concentration. Meanwhile, it is easy to distinguish FL intensity of 10 nM Cu^2+^ from 0 nM Cu^2+^, therefore, the limit of quantification (LOQ) for Cu^2+^ was set to 10 nM. Their corresponding FL intensities at the maximum emission peak were also recorded. Calibration curve (Fig. 4B, inset) showed that the FL intensity is proportional to the logarithm of Cu^2+^ concentration in the range of 10 nM to 100 μM with a linear equation of FL intensity = −577.22 (lg Cu^2+^) + 3560.34 (*R*^2^ = 0.9892).

**Fig. 4:**
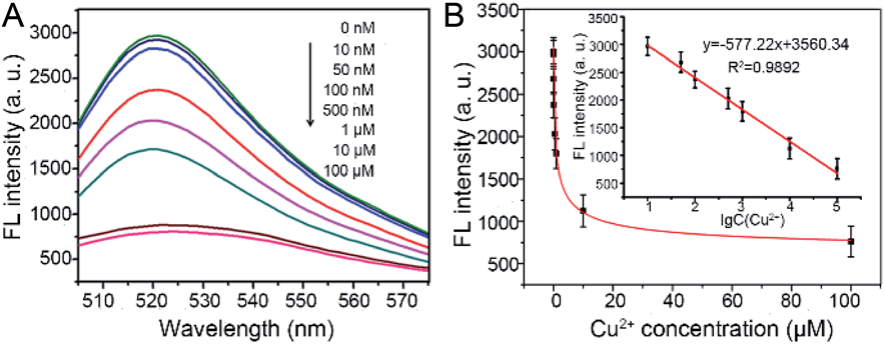
The sensitivity of this assay for Cu^2+^ detection under optimal experimental conditions. (A) Fluorescence spectra of solutions with different concentrations of Cu^2+^. (B) Plots of FL intensity *vs.* Cu^2+^ concentration. Inset: calibration curve of FL intensity *vs.* the logarithm of Cu^2+^ concentration. The error bars represent the standard deviation of three independent measurements.

To examine the specificity of this Cu^2+^ detection system, a set of metal ions, including K^+^, Na^+^, Pb^2+^, Hg^2+^, Ba^2+^, Cd^2+^, Sr^2+^, Fe^2+^, Mg^2+^, Co^2+^, were tested as controls. Besides, a mixture of Cu^2+^ (10 μM) with all competing metal ions (each 100 μM) was also tested as a control (donated as Mix). As shown in Fig. S3A,† 10 μM Cu^2+^ can cause a remarkable decrease of FL intensity, while neither one of the competing metal ions can cause the significant decrease of FL intensity. Meanwhile, the coexistence of other metal ions did not apparently affect the results for Cu^2+^ detection. These results demonstrate that our strategy exhibits an excellent selectivity to Cu^2+^ over other competing metal ions, which attributes to the high specificity of click chemistry in the complex biological samples.^26^

As we known, favorable biocompatibility is an important requisite for any nanomaterial used in intracellular imaging.^27^ To study the cytotoxicity of GDY NSs, three types of human cell lines, including HeLa, HepG2, 293T cell lines were tested systematically. As shown in Fig. S3B,† the viability of all selected cells was higher than 82% after incubation with high concentration (100 μg mL^−1^) of GDY NSs for 24 h, indicating the low toxicity and good biocompatibility of GDY NSs.^28,29^ Similar results were obtained by using AO/PI to stain the living and dead cells (Fig. S4†). To expand the potential biological application of this Cu^2+^ detection system, we used it to track endogenous Cu^2+^ in HepG2 cells. After incubation with Cu^2+^ for 2 h, an obvious fluorescence reduction was observed in HepG2 cells which were pre-incubated with both GDY NSs and N_3_-dsDNA-FAM (Fig. S3C and D†). These results demonstrate the potential of this assay for sensing Cu^2+^ in the living cells.

As mentioned above, detection of Cu^2+^ in human urine is an effective way for screening Wilson's disease at early stage.^30^ For this reason, the practical application of this Cu^2+^ detection system in human samples was conducted. Recovery experiments were performed by spiking different concentrations of Cu^2+^ (the final concentrations are 1 μM, 10 μM, and 100 μM) into diluted human urine sample (filtered with a 0.22 μm filter membrane before use). And the original Cu^2+^ concentration can not be detected in the diluted human urine samples using this method. The detection results are shown in Table S2.† The low recovery rate (73.8–86.23%) may be attribute to the influence of impurities in the urine on the test results. Our future work will focus on how to reduce the influence of real samples to the test results.

In this work, we have developed a novel 2D GDY-based Cu^2+^ detection system with high sensitivity and specificity, which is technically simple, easily manipulated, and cost-effective for practical applications. The LOQ for Cu^2+^ is 10 nM under optimal conditions with an acceptable recovery rate. Besides, the high specificity of the Cu^+^-catalyzed click reaction endows this sensor with an excellent selectivity against the interference of other metal ions. Furthermore, its ability to detect Cu^2+^ in living cells and human body fluids was also demonstrated, which suggests its potential for screening Cu^2+^-related diseases. Compared with previous studies on GDY NSs, this work not only provides a new method for accurate metal ion detection using GDY NSs as the fluorescence quenching platform, but also, opens up the investigations of the modified GDY NSs through butadiyne groups for detection other targets including miRNAs, DNAs, proteins, and small molecule. However, current understanding of the biocompatible of GDY NSs is extremely limited. GDY NSs tend to adsorb biomolecules in biosystems, which may prevent it from the click reaction and the FRET process. Much deeper work is needed for reducing the influence of biomolecules in body fluids on the test results.

## Conflicts of interest

There are no conflicts of interest to declare.

## Supplementary Material

RA-011-D0RA08595B-s001
