# Supplementary material for: Graphdiyne nanosheets as a platform for accurate copper(ii) ion detection via click chemistry and fluorescence resonance energy transfer
Source: RSC Adv. 2021 Jan 28;11(10):5320–4. doi: 10.1039/d0ra08595b (PMC8694639; doi:10.1039/d0ra08595b)
Supplement: RA-011-D0RA08595B-s001 [file RA-011-D0RA08595B-s001.pdf]

Electronic Supplementary Material (ESI) for RSC Advances.
This journal is © The Royal Society of Chemistry 2020

Supporting Information

Graphdiyne Nanosheets as a Platform for Accurate Copper (II) Ions Detection via Click Chemistry and fluorescence resonance energy transfer

Chenchen Ge,‡[ac] Jiaofu Li,‡[b] Dou Wang,‡[d] Kongpeng Lv,[a] Quan Liu,[a] Yan Shen,[a] Xiaoqing Zhuang,[a] Wankun Luo,[a] Zongze Wu,[a] Yuhua Zhang,[a] Lulin Shi,[a] Liping Liu,*[a] Shiyun Bao,*[a] Han Zhang*[b]

[a] *Department of Hepatobiliary and Pancreatic Surgery, The Second Clinical Medical College, Jinan University (Shenzhen People's Hospital), Shenzhen, 518020, China.*

E-mail: bao.shiyun@szhospital.com; liu.liping@szhospital.com

[b] *Institute of Microscale Optoelectronics, Collaborative Innovation Centre for Optoelectronic Science & Technology, Key Laboratory of Optoelectronic Devices and Systems of Ministry of Education and Guangdong Province, Guangdong Laboratory of Artificial Intelligence and Digital Economy (SZ), Shenzhen University, Shenzhen, 518060, PR China. E-mail: hzhang@szu.edu.cn.*

[c] *College of Health Science and Environmental Engineering, Shenzhen Technology University, Shenzhen, 518118, China*

[d] *Department of Biomedical Engineering, Southern University of Science and Technology, Shenzhen, 518055, China.*

‡ Chenchen Ge, Jiaofu Li and Dou Wang contributed equally to this work.

Experimental Procedures

Reagents. Sodium ascorbate (SA) was purchased from Shanghai Aladdin Reagent Co., Ltd. (Shanghai, China). Copper sulfate, potassium chloride, and other metal ions were purchased from Guangzhou Chemical Reagent Factory (Guangzhou, China). PBS (10 mM, pH 7.4), fetal bovine serum, Dulbecco's modified Eagle medium (DMEM), trypsin-EDTA, and penicillin/streptomycin (P/S) were purchased from Gibco Life Technologies. Acridine orange/propidium iodide (AO/PI) assay kit was obtained from Logos Biosystems (South Korea). Cell Counting Kit-8 (CCK-8) was purchased from TransGen Biotech Co., Ltd. (Beijing, China). Lipofectamine® 2000 was purchased from Life Technologies (California, USA). HeLa, HepG2, and 293T cells were purchased from ATCC (American Type Culture Collection). Human urine samples were obtained from healthy volunteers at Shenzhen People's Hospital. All experiments were performed in accordance with the Guidelines of Medical Ethics Committee of Shenzhen People's Hospital. Informed consents were obtained from human participants of this study. Oligonucleotides purified by HPLC were synthesized by Shanghai Sangon Biotechnology Co. Ltd. (Shanghai, China) and listed in **Table S1**. SsDNA1 and cDNA1 hybridize to form dsDNA. Ultrapure water (18.25 MΩ cm^{-1}) was used to prepare all of the solutions.

Table S1. The sequences used in this experiment.

Name	Sequence
ssDNA1	N_3-5'-TTTTTT-3'-FAM
cDNA1	5'-AAAAAA-3'
ssDNA2	N_3-5'-ATGCGATTGCCGTCATGGGTCGCCTGCATG -3'-FAM

Preparation of GDY NSs. GDY powder was synthesized according to the previously published procedure.[1,2] Briefly, the monomer of hexaethynylbenzene was synthesized by adding tetrabutylammonium fluoride into a tetrahydrofuran solution of hexakis-[(trimethylsilyl)ethynyl] benzene for 10 min at 8℃. The GDY was successfully grown on the surface of copper foil in the presence of pyridine by a cross-coupling reaction of the monomer of hexaethynylbenzene for 72 h at 60℃ under a nitrogen atmosphere. In the process of GDY formation, the copper foil severs

as not only the catalyst for the cross-coupling reaction but also the substrate for the growth of GDY film. The copper foils covered by GDY were first washed with acetone followed by hot (80℃) DMF under sonication for 1 hour to obtain black solid. The black solid was refluxed at 100℃ for 2 h in 4 M sodium hydroxide, 6 M hydrochloric acid, 4 M sodium hydroxide solution, respectively, to remove the impurities and copper residue. The product was collected by centrifugation, washing with hot DMF (80℃), hot ethanol (70℃), and then dried to give pure GDY. GDY powder was dispersed into Milli-Q water followed by exfoliation under sonication for 10 days (12 h each day) to prepare GDY NSs.

Instrumentation. The TEM and HR-TEM images were taken on the FEI Tecnai G2 F30 transmission electron microscope at an acceleration voltage of 300 kV. XPS spectra were collected by an X-ray photoelectron spectroscopy (ESCALAB 250, Thermo Fisher) instrument. Fourier Transform Infrared Spectra were recorded by Thermo Scientific Nicolet iS 50 spectrometer. Raman spectra was performed on a high-resolution confocal Raman microscope (HORIBA LabRAM HR800) at room temperature (RT). Zeta potential was performed by Malvern Mastersizer 2000 (Zetasizer Nano ZS90, Malvern Instruments Ltd., UK). The data was gained with the average of three times. The fluorescence intensities of the solution were recorded using the multimode microplate reader (Spark 10M, Tecan, Switzerland).

Experimental procedure for detecting Cu^{2+}. A typical procedure for assaying Cu^{2+} is as follows. N_3-dsDNA-FAM was prepared by mixing equal moles of DNA1 and cDNA1 in PBS, the mixture was heated to 95℃ for 5 min, then slowly cooled down to RT and stored at 4℃ before use. GDY was dissolved in PBS by probe ultrasound for 5 h and mixed with N_3-dsDNA-FAM to a final concentration of 10 μg/mL and 10 nM, respectively. Cu^{2+} with various concentrations (0, 0.01, 0.05, 0.1, 0.5, 1, 10, and 100 μM) and SA (500 μM) were added to each reaction at a final volume of 200 μL, and the mixture was shaken gently for 2 h at RT. The solutions were transferred into a 96-well microtiter plate, and fluorescence intensities of the solution in each well were recorded using the multimode microplate reader (Spark 10M, Tecan, Switzerland). The excitation wavelength and emission wavelength are 475 nm and 518 nm, respectively.

Cytotoxicity of GDY NSs. The cytotoxicity of GDY NSs was investigated by the standard CCK-8 assay and AO/PI fluorescent assay. HeLa, HepG2, and 293T cells were cultured in DMEM supplemented with 10% FBS, and 100 U/mL P/S, the cells were maintained at 37 °C in a humidified atmosphere with 5% CO_2. Briefly, HeLa, HepG2, and 293T cells were seeded in a 96 well plate (1.2×10^4 per well) and cultured for 24 h to allow attachment. Cells were then incubated with different concentrations of GDY (1 μg/mL, 10 μg/mL, 50 μg/mL, 100 μg/mL) in medium for 24 h. The medium was replaced with 100 μL medium containing 10 μL of CCK-8 solution and incubated for additional 1h. The absorbance was measured at 450 nm using the multimode microplate reader (Spark 10M, Tecan, Switzerland). For the AO/PI fluorescent assay, after the cells were incubated with different concentrations of GDY (1 μg/mL, 10 μg/mL, 50 μg/mL, 100 μg/mL) in medium for 24 h, AO (live cells, green fluorescence)/PI (dead cells, red fluorescence) were used to co-stain the cells for 5 min, the images were visualized using a Leica DMi8 inverted microscope connected to a DFC 7000T camera.

Cell fluorescence imaging. HepG2 cells were seeded in a 96 well plate (1.2×10^4 per well) and cultured for 24 h to allow attachment. Cells were washed twice with PBS and then incubated with 1 nmol N_3-dsDNA-FAM/Lipofectamine® 2000 complexes and 10 μg/mL GDY (in the culture medium) for 4 h at 37 °C. SA and Cu^{2+} were added to a final concentration of 10 μM for another 2h. The fluorescence images were observed and recorded using a Leica DMi8 inverted microscope connected to a DFC 7000T camera.

Recovery experiment. To detect free and spiked Cu^{2+} (the final concentrations are 1 μM, 10 μM, and 100 μM) in urine sample, 2 μL sample, 2 μL GDY solution, and 2 μL N_3-dsDNA-FAM were added to 188 μL PBS, the final concentrations for GDY and N_3-dsDNA-FAM were 10 μg/mL and 10 nM, respectively. Then SA (2 μL) was added to reach a final concentration of 100 μM and incubated for 2 h. All the results were recorded using the multimode microplate reader.

Supplementary Figures

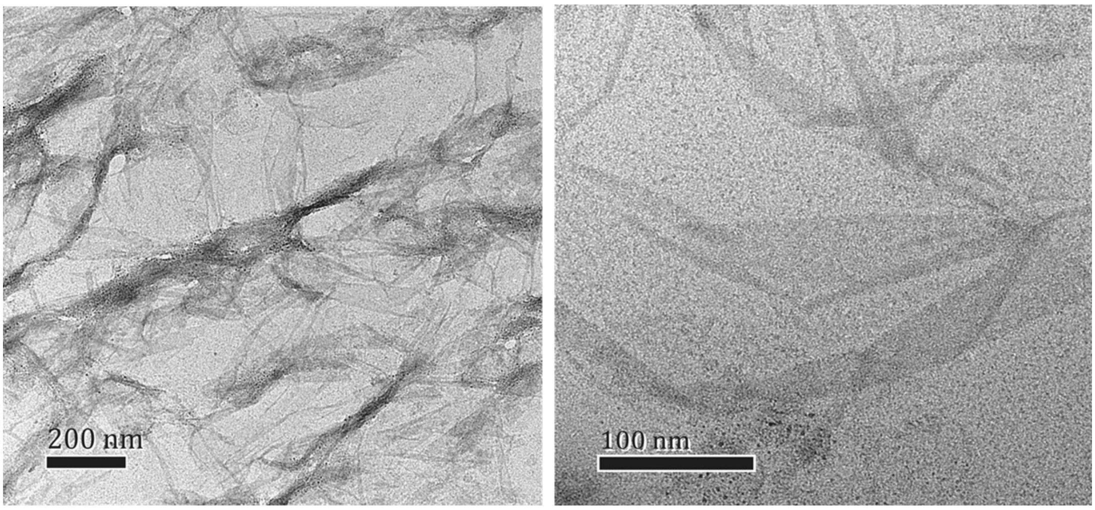

Figure S1. TEM images of GDY NSs.

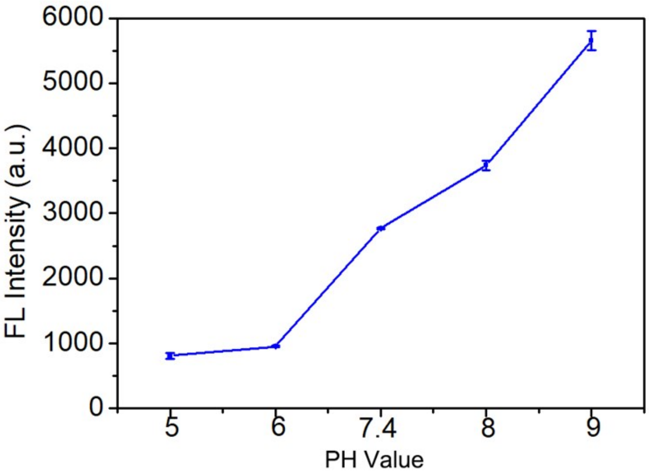

Figure S2. FL intensity of N_3-dsDNA-FAM under different pH conditions. The concentration of N_3-dsDNA-FAM was 10 nM.

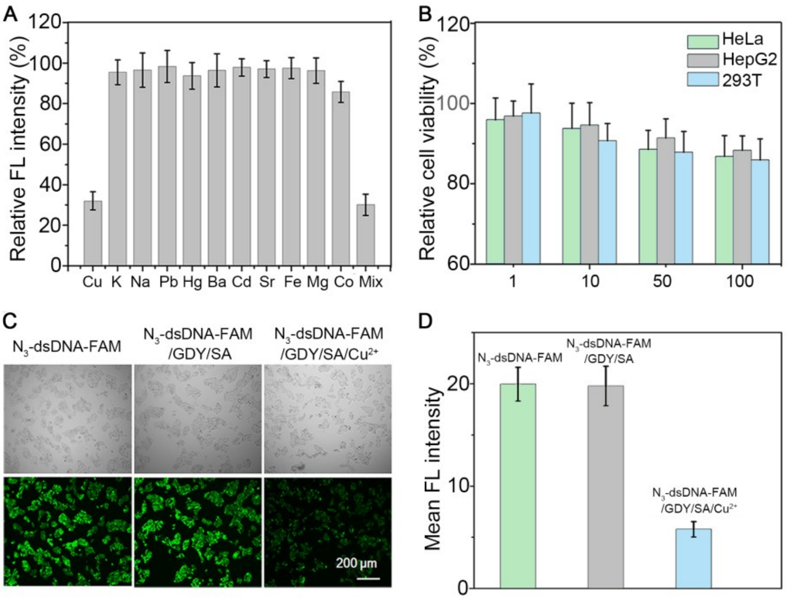

Figure S3. The specificity and cytological experiments of this assay. (A) The difference in relative FL intensity between Cu^{2+} (10 μM) and other competing metal ions (each 100 μM). Relative FL intensity is F/F0, where F is the FL intensity of metal ions with different concentrations and F0 is the FL intensity without Cu^{2+}. (B) The viability of HeLa, HepG2, and 293T cells after exposure to different concentrations of GDY NSs for 24 h. (C) The fluorescence images of HepG2 cells incubated with N_3-dsDNA-FAM, N_3-dsDNA-FAM/GDY/SA, and N_3-dsDNA-FAM/GDY/SA/Cu^{2+}. (D) Quantifed mean FL intensity inside cells from (C). The error bars represent the standard deviation of three independent measurements.

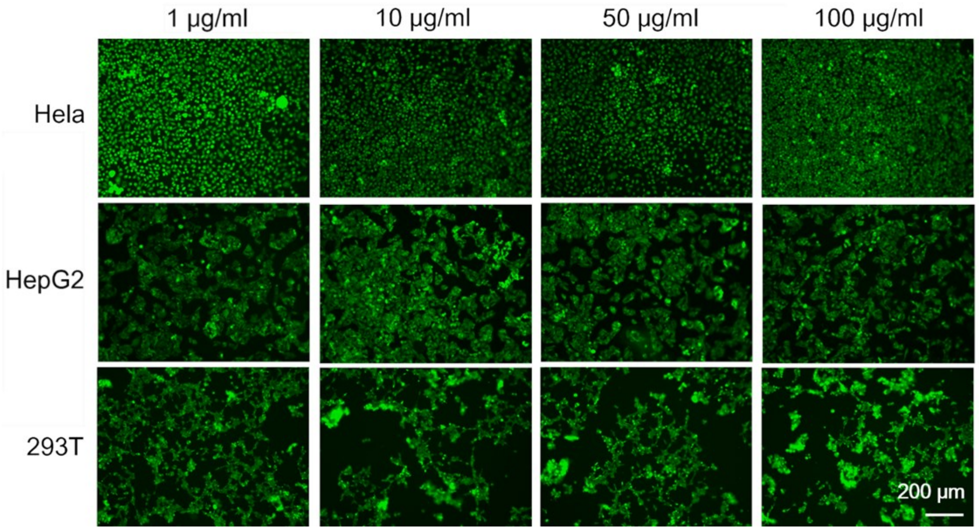

Figure S4. AO/PI staining of HeLa, HepG2, and 293T cells after incubation with different concentrations of GDY NSs for 24 h.

Table S2. Recovery experiments for Cu^{2+} detection in human urine samples.

Sample	Cu^{2+} (μM) (n=3)		Recovery
	Spiked	Detected	(%)
Human Urine	1	0.75±0.09	75.0
	10	7.38±1.06	73.8
	100	86.23±6.96	86.23

References

1. G. Li, Y. Li, H. Liu, Y. Guo, Y. Li and D. Zhu, Chemical communications, 2010, 46, 3256-3258.

2. H. Qi, P. Yu, Y. Wang, G. Han, H. Liu, Y. Yi, Y. Li and L. Mao, Journal of the American Chemical Society, 2015, 137, 5260-5263.
